# Role of Transforming Growth Factor Beta in Peripheral Nerve Regeneration: Cellular and Molecular Mechanisms

**DOI:** 10.3389/fnins.2022.917587

**Published:** 2022-06-13

**Authors:** Zhiqian Ye, Junbin Wei, Chaoning Zhan, Jin Hou

**Affiliations:** ^1^The First School of Clinical Medicine, Southern Medical University, Guangzhou, China; ^2^Department of Stomatology, Nanfang Hospital, Southern Medical University, Guangzhou, China

**Keywords:** transforming growth factor beta, peripheral nerve regeneration, Schwann cell, macrophage, peripheral nerve injury (PNI)

## Abstract

Peripheral nerve injury (PNI) is one of the most common concerns in trauma patients. Despite significant advances in repair surgeries, the outcome can still be unsatisfactory, resulting in morbidities such as loss of sensory or motor function and reduced quality of life. This highlights the need for more supportive strategies for nerve regrowth and adequate recovery. Multifunctional cytokine transforming growth factor-β (TGF-β) is essential for the development of the nervous system and is known for its neuroprotective functions. Accumulating evidence indicates its involvement in multiple cellular and molecular responses that are critical to peripheral nerve repair. Following PNI, TGF-β is released at the site of injury where it can initiate a series of phenotypic changes in Schwann cells (SCs), modulate immune cells, activate neuronal intrinsic growth capacity, and regulate blood nerve barrier (BNB) permeability, thus enhancing the regeneration of the nerves. Notably, TGF-β has already been applied experimentally in the treatment of PNI. These treatments with encouraging outcomes further demonstrate its regeneration-promoting capacity. Herein, we review the possible roles of TGF-β in peripheral nerve regeneration and discuss the underlying mechanisms, thus providing new cues for better treatment of PNI.

## Introduction

Unlike the nerves in the central nervous system, peripheral nerves have significant potential to regenerate and repair after injuries (Clements et al., 2017). Despite numerous surgical advancements and improved understanding of nerve regeneration, adequate restoration of function remains an unfulfilled clinical goal (Fregnan et al., 2012; Palispis and Gupta, 2017). Even in an optimal surgical situation of direct nerve repair, a large proportion of patients with peripheral nerve injury (PNI) failed to achieve satisfactory motor and sensory recovery (Ruijs et al., 2005; Paprottka et al., 2013; Kemp et al., 2017). This often results in sensory and motor deficits, such as partial or permanent paralysis, debilitating neuropathic pain, numbness of the limb, cold intolerance and dysesthesias, all of which severely impact the quality of life (Kemp et al., 2017; Palispis and Gupta, 2017). Therefore, to achieve optimal functional recovery, it is important to elucidate molecular mechanisms of nerve regeneration and focus on factors that will enhance and improve regenerative processes.

Challenges associated with meaningful functional recovery after PNI primarily include the rate of regeneration, specificity of regeneration, segmental nerve defects and degeneration of the target-end organs (Palispis and Gupta, 2017). The search for solutions to these obstacles starts with understanding the pathophysiological processes of peripheral nerve regeneration. Neural repair is a complex biological process, including the clearance of myelin debris (Rotshenker, 2011), the formation of bands of Büngner that provide physical guidance for regrowing axons (Min et al., 2021), and the synthesis of neurotrophic factors, extracellular matrix (ECM) and cell-adhesion molecules (Luo et al., 2012; Pereira et al., 2012; Carvalho et al., 2020). In addition, the activation of the neuronal intrinsic growth capacity (Chandran et al., 2016), blood nerve barrier (BNB) permeability (Mizisin and Weerasuriya, 2011) and the inflammation level of wound microenvironment (Molnár et al., 2022; Yu et al., 2022) also affect the outcome of neural regeneration. Among the various types of cells that participate in the regenerative process, Schwann cells (SCs) and macrophages are considered to have the most prominent roles (Chen et al., 2015c; Jessen et al., 2015; Kubiak et al., 2020). Potential therapies to improve functional recovery should target factors that guide biological responses as well as structural and cellular components in peripheral nerve regeneration toward a pro-regenerative trend.

Transforming growth factor-β (TGF-β) proteins are multifunctional cytokines. TGF-β is a well-studied factor involved in various physiological processes like cell proliferation, differentiation, wound healing and immune response. It is also implicated in the pathogenesis of several diseases such as connective tissue diseases, skeletal diseases, fibrosis, and cancer progression (Morikawa et al., 2016). They regulate the development and functions of the nervous system and their neuroprotective roles are increasingly recognized (Dobolyi et al., 2012; Bielmeier et al., 2022; Lee et al., 2022). During the regenerative process of peripheral nerves, TGF-β is released by macrophages (Sulaiman and Gordon, 2009), SCs (Li et al., 2015; Clements et al., 2017), fibroblasts (Clements et al., 2017), and the injured nerves (van Rossum et al., 2008; Li et al., 2015) and is abundantly present in the nerve bridge microenvironment (Xiao et al., 2014; Clements et al., 2017), implying its vital involvement in the process. Current evidence highlights the pro-regenerative effects and complex roles of TGF-β in many biological processes and components that are essential for nerve regeneration. In this review, we summarize the involvement of TGF-β in peripheral nerve regeneration and discuss the possible underlying mechanisms. We also summarize the application of TGF-β in the experimental treatment of PNI, explore its clinical translation and provide new cues for potential therapeutics to manage PNI.

## Pathophysiological Process of Peripheral Nerve Injury and Regeneration

Based on the extent of damage to the connective tissues and the axons of the nerve, PNI was first classified by Seddon into three categories, namely neurapraxia, axonotmesis, neurotmesis, and by Sunderland into five degrees (I–V) (Seddon, 1943; Sunderland, 1951). The mildest form called neurapraxia, is characterized by local demyelination without axon or connective tissue lesion. In addition to demyelination, axonal lesion but with preserved connective tissue is termed axonotmesis. The most severe form of injury is neurotmesis, which is a full transection of axons and connective tissue (Menorca et al., 2013). When the gravity of the injury reaches the level of axonotmesis–Sunderland III, where the continuity of axon and myelin is disrupted, Wallerian degeneration occurs and consequently leads to incomplete recovery (Carvalho et al., 2020).

Wallerian degeneration is the first process after nerve injury and occurs in the distal end of the damage 24–48 h after the injury. During this process, the distal axons, the adjacent myelin and other axonal components start to degenerate to clear the undesired debris (Conforti et al., 2014). Removal of degenerated myelin is critical for nerve repair as myelin inhibits the regeneration of severed injured nerves (Vargas et al., 2010; Jessen and Mirsky, 2019). Myelin clearance is a two-phase process mainly completed by macrophages and SCs. The first phase of myelin clearance occurs 5–7 days after injury, wherein SCs play a major part by breaking down their redundant myelin sheaths. It is suggested that during the initial 24 h after injury, SCs degrade myelin basic protein through a matrix metalloproteinase (MMP)-dependent pathway (Austin and Moalem-Taylor, 2010). Approximately 50% of the myelin is removed during this first phase. The second phase is dominated by macrophages, including tissue-resident macrophages and blood-derived macrophages (Jessen and Mirsky, 2016). Tissue-resident macrophages increase and are activated to participate in the initial removal of debris before the influx of hematogenous macrophages. Hematogenous macrophages begin the infiltration at 2–3 d after injury and peak between 7 and 14 days (DeFrancesco-Lisowitz et al., 2015). During this process, myelin fragments, and various chemoattractants released by SCs and tissue-resident macrophages help recruit hematogenous macrophages to remove the remaining debris (Rosenberg et al., 2012; Chen et al., 2015c).

While Wallerian degeneration takes place in the distal stump and regrowing axons extend from the proximal stump, SCs experience a series of injury-induced changes that involve both the loss and gain of cellular phenotypes, namely reprogramming or transdifferentiation (Namgung, 2014; Chen et al., 2015c; Clements et al., 2017). Reprogramming confers the property of plasticity on SCs and is a vital basis of peripheral neural regeneration (Jessen et al., 2015). Features of repair SCs mainly include dedifferentiation from a myelinated to a progenitor-like cell (Jessen and Mirsky, 2019), secretion of neurotrophic factors to promote axonal survival (Pandey and Mudgal, 2021), production of cytokines to recruit immune cells (Qu et al., 2021) and morphological changes (Jessen and Mirsky, 2016). Following cellular reprogramming, repair SCs proliferate and sort into cords and extend longitudinally to form bands of Büngner to help guide axonal regrowth (Arthur-Farraj et al., 2012). Bands of Büngner are crucial in controlling the directionality and speed of axonal regeneration across the nerve gap (Min et al., 2021). Directionality and rate are important elements of successful recovery (Bearce et al., 2015). Current research evidence shows that during the initial 5 days after mouse sciatic nerve transection injury, when regenerating axons regrow in front of migrating SCs as axons regenerate much before SC migration begins, axons lack directionality and form bundles at the regeneration front, thus impeding regeneration. Then, on day 7, SCs migrate in front of axons and form SC cords, leading axons to grow much more rapidly and directionally toward the distal stump (Chen et al., 2019).

Besides clearance of debris and formation of bands of Büngner, SCs also help create a microenvironment that favors axonal regeneration and recruitment of macrophages. They are vital sources of endogenous neurotrophic factors, promoting neuronal survival and axonal regeneration after acute or chronic nerve injuries (Xu et al., 2010). Previous research on rats suggests that the loss of neurotrophic support by SCs in the distal stump of injured nerves leads to fewer mature axons reaching their targets, which translates into insufficient functional recovery. Additionally, the recruitment of macrophages in the distal nerve stump is partly attributed to the chemokines produced by SCs (Sulaiman and Dreesen, 2014). Many studies have found that macrophages, particularly M2 macrophages play an important role in peripheral nerve repair (Chen et al., 2015b; Lv et al., 2017; Ehmedah et al., 2019; Huang et al., 2020). By releasing anti-inflammatory cytokines, they help create a favorable environment for axonal regrowth (Rotshenker, 2011; Liu F. et al., 2019). M2 macrophages efficiently clear the myelin debris for their higher expression level of phagocytosis-associated molecules (Zizzo et al., 2012; Healy et al., 2016). Interestingly, M2 macrophages can interact with SCs to enhance their capacity to promote axonal regeneration (Zhan et al., 2015). At the site of injury, TGF-β is not only an activator of M2 macrophages but also a vital effector of their various regeneration-promoting functions. TGF-β is known for its pleiotropic functions. The activation of Smad-dependent and -independent pathways, and the interaction of Smads with other transcription co-modulators, greatly extend the biological response of TGF-β in the nervous system and different stages of peripheral nerve regeneration (Clark and Coker, 1998; Kandasamy et al., 2014; Abarca-Buis et al., 2021).

## Role of Transforming Growth Factor-β in Peripheral Nerve Regeneration

### Schwann Cell Reprogramming

Following PNI, adult SCs are reprogrammed into a repair-competent state (Min et al., 2021). As mentioned above, phenotypic changes of repair SCs mainly include dedifferentiation (Jessen and Mirsky, 2019), secretion of neurotrophic factors (Pandey and Mudgal, 2021), production of cytokines to recruit immune cells (Qu et al., 2021), and changes in morphology (Jessen and Mirsky, 2016). After nerve injury, it is shown that a large amount of TGF-β is secreted by macrophages (Sulaiman and Gordon, 2009), SCs (Li et al., 2015; Clements et al., 2017), fibroblasts (Clements et al., 2017), and the neurons (van Rossum et al., 2008; Li et al., 2015) at the site of injury (Jeon and Huxlin, 2020). Meanwhile, the expression level of TGF-β receptor is significantly upregulated in SCs and neurons (Fregnan et al., 2012). Additionally, TGF-β was found to regulate the proliferation and differentiation of SCs from the quiescent myelinating phenotype to the proliferative activated state (Sulaiman and Gordon, 2002; Li et al., 2015; Clements et al., 2017). Thus, TGF-β appears to exert potent effects during SC reprogramming.

Dedifferentiation is an important part and an indicator of the beginning of SC reprogramming. The capacity of TGF-β to promote SC dedifferentiation is supported by current findings. In purified SCs, treatment with TGF-β1 induces inhibition of myelin P0 expression, indicating the blockage of the myelination process (Fregnan et al., 2012). Using a co-culture system that includes SCs and dorsal root ganglion neurons, TGF-β was found to block the expression of myelin-related molecules such as galactocerebroside, P0, myelin-associated glycoprotein and myelin basic protein. In contrast, the expression of molecules characteristic of mature non-myelinating SCs (Remak SCs) including neural-cell adhesion molecule (N-CAM), L1-cell adhesion molecule (L1-CAM), and nerve growth factor (NGF) receptor, was maintained. Notably, the expression of the glial fibrillary acidic protein, a molecule characteristic of Remak SCs, was increased 10-fold during coculture with TGF-β (Guénard et al., 1995), indicating the dedifferentiated state of SCs.

During the reprogramming process, SCs produce pro-regenerative factors, such as the neurotrophin receptor, p75, neuregulin, and the potent glial cell line-derived neurotrophic factor (GDNF) (Kim et al., 2017). These factors are considered essential as it has been shown in rats that enough mature axons do not reach their targets because of the loss of neurotrophic support by SCs in the distal stump of injured nerves (Sulaiman and Dreesen, 2014). TGF-β can significantly enhance the growth-promoting effects of neurotrophic factors released by SCs. Among various neurotrophic proteins, GDNF is considered a potent factor, and interestingly, the effects of GDNF have been found to require the presence of TGF-β both *in vitro* and *in vivo* (Schober et al., 1999; Sulaiman, 2016). TGF-β can help SCs recruit immune cells as well. When cocultured with TGF-β, SCs significantly elevate the expression levels of Interleukin (IL)-1β, IL-6, and IL-12, thus playing a role in leukocyte recruitment and further clearance of the myelin debris (Ozaki et al., 2008; Liu P. et al., 2019). Notably, cell adhesion molecules are considered critical for axonal regeneration, and TGF-β can increase their expression in SCs. Dedifferentiated and non-myelinating SCs express high levels of N-CAM and L1-CAM, whereas mature and myelinated SCs express low levels of them. L1-CAM and N-CAM, expressed by SCs, belong to the immunoglobulin-like CAM family. L1-CAM on SCs can bind to N-CAM expressed on axons and N-CAM on SCs can bind to axonal L1-CAM to promote axonal regeneration (Colombo and Meldolesi, 2015). At the transcription level, TGF-β1 upregulates mRNA of N-CAM and L1-CAM (Awatramani et al., 2002).

Repair SCs exhibit both bipolar and unipolar morphologies, which enhances their migratory capacity and thus contributes to the formation of bands of Büngner (Wang et al., 2012; Kim et al., 2013). Studies have supported the potential involvement of TGF-β in SC morphological changes. It was shown that TGF-β induces the polarization of SCs and thus facilitates the formation of bands of Büngner (Ribeiro-Resende et al., 2009). Exposure to TGF-β1 rapidly and significantly altered the cellular morphology of SCs, making them spread and extend multiple processes (Chandross et al., 1995). This can be attributed to TGF-β1 upregulating β1 integrin expression, thus enabling the SCs to more sensitively detect and respond to the local cues in the environment (Rosner et al., 2005).

### Clearance of Myelin Debris

Because of the inhibitory effects of myelin on neural regeneration, rapid clearance of myelin is of vital importance (Rotshenker, 2011; Carvalho et al., 2020). SCs initiate the first phase of myelin clearance (Gomez-Sanchez et al., 2015). MMPs may play a crucial role in this process. It is suggested that SCs degrade myelin basic protein through an MMP-dependent pathway in the first 24 h after injury (Austin and Moalem-Taylor, 2010). Notably, TGF-β1 regulates the activities of MMPs in SCs by increasing mRNA and protein expression of MMP-2 and MMP-9 (Muscella et al., 2020), thereby promoting myelin clearance during the early stage of Wallerian degeneration.

Later, the removal of myelin is dominated by macrophages (Boissonnas et al., 2020). The process mainly comprises recruitment of and phagocytosis by macrophages (Rosenberg et al., 2012; Chen et al., 2015c). Current evidence suggests that TGF-β promotes the recruitment of macrophages after peripheral nervous system (PNS) injuries. Brief treatment with TGF-β1 was found to induce directed macrophage migration. In response to TGF-β1, Ras homolog A is activated and its activity is relevant to cell migration *via* the production of chemokines, including macrophage inflammatory protein and monocyte chemoattractant protein-1 (Kim et al., 2006). Monocytes consistently express high-affinity TGF-β receptors on their surface (Morikawa et al., 2016). More specifically, TGF-β was observed to recruit more M2 macrophages during wound repair (Qin et al., 2019).

TGF-β treatment was shown to upregulate the phagocytosis-associated receptors of the infiltrated macrophages, thus promoting their ability to phagocytose myelin debris (Liu F. et al., 2019). Macrophages were found to express high levels of phagocytosis-associated molecules after their rapid invasion into peripheral nerves (van Rossum et al., 2008). Macrophages phagocytose myelin differently from phagocytosis of other components. This notion is supported by the finding that macrophages from patients with multiple sclerosis display a reduced ability to phagocytose human myelin but not red blood cells, which could be attributed to their lower levels of the phagocytic tyrosine kinase receptor, MerTK (Healy et al., 2017). TGF-β was found to directly elevate the expression of MerTK in macrophages, thus enhancing the phagocytosis of myelin (Healy et al., 2017). After exposure to TGF-β, myelin digestion is reported to be significantly enhanced in microglia, and MerTK is among the most upregulated differentially expressed genes in these cells. Given that the central nervous system myeloid population comprises endogenous microglia and monocyte-derived macrophages from peripheral blood circulation and that there is phenotypic convergence between two types of cells (Grassivaro et al., 2021), this result and the associated mechanism are of significant value in the context of PNI (Healy et al., 2016). TGF-β can also indirectly promote macrophages to phagocytose myelin by polarizing macrophages to the M2 phenotype. The polarization of macrophages evidently affects the expression of MerTK. Current evidence shows that MerTK expression is restricted to anti-inflammatory M2c macrophages among macrophage subsets (Zizzo et al., 2012; Zizzo and Cohen, 2018). TGF-β is consistently capable of polarizing macrophages to the M2 phenotype as discussed in the next sections.

## Regulation of Injury/Regeneration Environment

### Macrophages Support the Growth-Supportive Functions of Schwann Cells

During Wallerian degeneration, SCs play a critical role in axon regeneration by creating a growth-supportive environment (Sulaiman and Dreesen, 2014). However, SCs cannot maintain this environment without the assistance of macrophages (Li et al., 2022). The pro-regenerative capacity of SCs and injured neurons deteriorates if nerve repair is delayed for more than 4–6 weeks (Sulaiman and Gordon, 2009). Interestingly, the deterioration of the regeneration-supportive function of SCs after 4 weeks coincides with the decline in the number of infiltrated macrophages (Sulaiman and Dreesen, 2014). Some studies suggest that macrophages are charged with stimulating the production of neurotrophic factors by non-neuronal cells (such as SCs) in the distal stump (Echeverry et al., 2013). A study found that macrophages, particularly the M2 phenotype, secrete microvesicles to elevate migration and proliferation of SCs, increase NGF and laminin production by SCs, thus enhancing neuronal maintenance and neural regeneration (Zhan et al., 2015). Taken together, these findings indicate that macrophages are essential for the promotive effects of SCs on nerve repair.

TGF-β is closely associated with the supportive effects of macrophages on SCs. Current evidence suggests that TGF-β is essential for the growth-supportive functions of SCs. It has been shown that TGF-β can activate SCs and sustain the non-myelinating, growth-promoting state and even reverse the incapacity of chronically denervated SCs to support axonal regeneration (Sulaiman and Gordon, 2009). Accordingly, the decline in the levels of TGF-β and other growth factors secreted by the activated macrophages is associated with the loss of response of SCs (Sulaiman and Dreesen, 2014). Macrophages have been widely acknowledged as important biological sources of TGF-β1 (Qin et al., 2018), which is applied in the context of PNI as well (Sulaiman and Dreesen, 2014), wherein TGF-β1 secreted by macrophages was found to induce the expression of several neurotrophic factors in SCs (Sulaiman et al., 2018). In addition, when placed in a silastic tube that bridged the gap between proximal and distal nerve stumps, chronically denervated SCs incubated with TGF-β reportedly promoted twice as many motoneurons to regenerate their axons as those not incubated with TGF-β (Sulaiman and Nguyen, 2016). Taken together, these findings provide strong evidence for the notion that the support of macrophages to SCs is essential to sustain axonal regeneration and TGF-β plays a critical role in this process.

**FIGURE 1:**
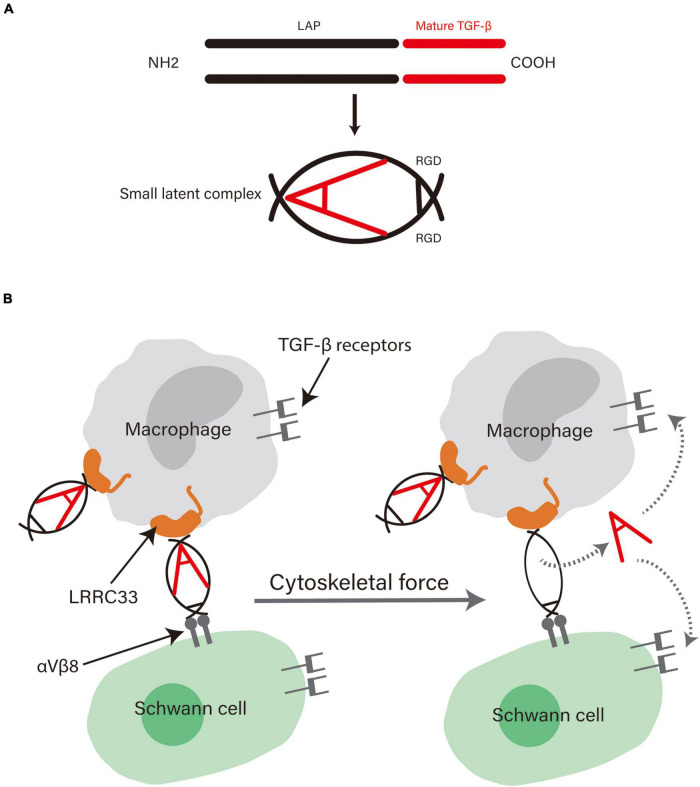
**(A)** The structure of pro-TGF-β. Pro-TGF-β is an inactive complex comprising a LAP and a mature cytokine. The LAP surrounds the mature cytokine and blocks its binding to receptors. Adapted from Travis and Sheppard (2014) and republished with permission from Annual Reviews, Inc. **(B)** The possible mechanism behind the role of TGF-β in the supportive effects exerted by macrophages on SCs. αVβ8 integrins on SCs can bind with high affinity to the Arg-Gly-Asp motif in the LAP and LRRC33 on macrophages bind to the other side of it. The physical force generated by contraction of the actin cytoskeleton of cells distorts the LAP and releases the cytokine, thus enabling high selectivity in TGF-β activation and cellular functions.

Some studies on the special way of TGF-β activation shed some light on the underlying mechanisms of TGF-β in supportive effects of macrophages on SCs (Figure 1). TGF-β is secreted as an inactive complex (termed the small latent complex) with an N-terminal latency associated peptide (LAP) and a C-terminal mature cytokine. The LAP region folds around the mature cytokine, thus blocking the access of TGF-β to its receptor (Travis and Sheppard, 2014). Pro-TGF-β is produced by macrophages on the cell surface and can be activated in a special way. The small latent complex can be anchored to the surface of macrophages *via* an association with leucine-rich repeat-containing protein 33 (LRRC33), a milieu molecule for TGF-β expressed on myeloid cells (Qin et al., 2018; Batlle and Massagué, 2019). Meanwhile, the complex binds to αVβ6 or αVβ8 integrin through the Arg–Gly–Asp integrin recognition motif present in the LAP. Upon cell contraction, the tension exerted by the cytoskeleton on integrins, which is resisted by the LRRC33 linked to the complex, distorts the LAP, releases the mature cytokine, and thus activates TGF-β (Batlle and Massagué, 2019). This interaction between cells bearing the milieu molecule and integrin-bearing cells enables great cellular selectivity and localization of TGF-β activation to the interface between the two kinds of cells (Qin et al., 2018). Interestingly, SCs carry a sufficient amount of integrins on their surface, including αVβ8 integrins, which can bind to the pro-TGF-β complex (Catignas et al., 2021). Taken together, the highly specific activation of TGF-β may be the underlying mechanism conferring the supportive effects macrophages exert on SCs, further demonstrating the critical role of TGF-β in the interaction between them.

### Modulation of the Extracellular Matrix

The ECM provides a physiological environment that supports the survival and development of axons and other supportive cells. For the ECM of SCs, the basal lamina is considered as a key component and mainly consists of collagen type IV, laminin and fibronectin (Gonzalez-Perez et al., 2013; de Luca et al., 2014). These components are upregulated following nerve injury to help form bands of Büngner and facilitate axonal regeneration (Carvalho et al., 2020). Many studies have shown that TGF-β modulates various cell adhesion molecules and the expression of ECM components in SCs, including laminin, collagen, fibronectin, tenascin, thrombospondin, and proteoglycans (Fu and Gordon, 1997; Clements et al., 2017).

Notably, the SC-derived basement membrane is regarded as a scaffold for promoting nerve regeneration, cell migration, enhancing contact and sorting to form bands of Büngner (La Fleur et al., 1996; Sulaiman and Gordon, 2009), and maintaining the physiological structure of nerve tissue. Following nerve injury, nerve tissue remodeling is associated with the balance between MMPs and their antagonist tissue inhibitor of metalloproteinases-1 (TIMP-1), and excessive MMPs can lead to degradation of the basement membrane. Nevertheless, even with high exposure to degrading enzymes, the SC-derived basement membrane is not degraded and maintains its integrity (La Fleur et al., 1996). TGF-β secreted by macrophages is shown to upregulate TIMP expression, thus protecting the collagen type IV in basement membrane against MMP9-induced degradation, and consequently promoting axon growth (La Fleur et al., 1996).

TGF-β can promote SCs migration by modulating ECM molecules. Tenascin C is an ECM component that promotes SC migration *via* β1 integrin-dependent pathway and its synthesis increases in fibroblasts with TGF-β1 stimulation (Zhang et al., 2016). Periostin, another ECM component, is secreted by SCs, particularly in migratory SCs (Sonnenberg-Riethmacher et al., 2015). Research has shown that TGF-β1 can increase the expression of periostin, thereby enhancing the migration of SCs and formation of bands of Büngner (Sonnenberg-Riethmacher et al., 2015). Additionally, periostin appears to be able to interact with other ECM components like collagen and tenascin-C, consequently playing a regulatory role in nerve fiber outgrowth (Pita-Thomas et al., 2017).

### Blood Nerve Barrier Permeability

The changes in the permeability of BNB during Wallerian degeneration create an essential environment for axon growth. In the first 2 weeks after nerve injury, BNB breaks down and this allows various blood factors and cells that enhance neural regeneration to enter the nerve (Fregnan et al., 2012). After the repair procedure, BNB integrity needs to be restored to stabilize the internal environment and maintain the homeostasis of peripheral nerves (Takeshita et al., 2020). TGF-β is suggested to play certain roles in altering BNB permeability.

The BNB is mainly localized in the microvessels of peripheral nerves within the endoneurium and comprises microvascular endothelial cells bound by tight junction proteins, pericytes attached to the outer side of the vascular endothelial cells, and the basement membrane covering these two types of cells (Kanda, 2013; Takeshita et al., 2020). Among these BNB components, current evidence suggests that TGF-β increases BNB permeability by acting on pericytes and the basement membrane. Pericytes are suggested to be the key regulator of the basement membrane of the BNB. The basement membrane plays an important role in maintaining the structure and function of the capillary vessels of the BNB (Shimizu et al., 2011). Studies have shown that with TGF-β1 incubation, although the pro-MMP-9 proteins in peripheral nerve pericytes are upregulated, TIMP-1 production is not affected, thus leading to degradation of the basement membrane (Shimizu et al., 2011). Similarly, TGF-β1 was observed to enhance MMP-2 expression in pericytes. In addition to degrading the basement membrane, MMPs can cleave non-ECM substrates, including the LAP of latent TGF-β1. This can promote increased release of the active form of TGF-β1, potentially creating a positive feedback loop that exacerbates BNB permeability (Brkic et al., 2015). Aside from pericytes, several other cells of the neurovasculature unit (e.g., endothelial cells) also contain TGF-β receptors and may thereby contribute to TGF-β-induced changes in BNB (Rustenhoven et al., 2016). However, some tight junction proteins, such as ZO-1 and occludin, which are also instrumental in maintaining BNB integrity, can be rescued by TGF-β addition (Reinhold and Rittner, 2017). ZO-1 is a tight junction component between adjacent perineurial cells and is also expressed in endoneurial vessels (Reinhold and Rittner, 2017, 2020). Studies have shown that TGF-β1 increases ZO-1 expression *via* the MEK/ERK signaling pathway in endothelial cells of the blood–brain barrier, reduces the permeability of the BNB and promotes perineurium formation in peripheral nerves (Morris et al., 2017). Furthermore, research has suggested that following PNI, monocyte chemoattractant protein-1 and other inflammatory mediators impair blood–spinal cord barrier permeability, whereas extrinsic TGF-β rescues the injury-induced downregulation of ZO-1, occludin and N-cadherin, with Smad2/3 pathway activated in endothelial cells (direct effects) and local inflammation alleviated (indirect effects) (Echeverry et al., 2011; Nakazaki et al., 2021). Taken together, these findings suggest that at different stages of nerve repair, TGF-β exerts effects on different targets of the BNB and regulates its permeability, thus contributing to the pro-regenerative microenvironment.

### Anti-inflammation

Although successful nerve repair requires pro-inflammatory cytokines to recruit immune cells for multiple purposes as discussed above, many studies suggest that inflammation is detrimental to nerve regeneration. For instance, the nerve bridge is considered to have an inflammatory environment that is unfavorable for axonal outgrowth (Clements et al., 2017). In a rat model of sciatic nerve crush injury, it was shown that the inflammatory nerve microenvironment impaired peripheral nerve maintenance and regeneration by influencing SC repair processes (Büttner et al., 2018). TGF-β is a potent anti-inflammatory cytokine. A study evaluated the neural regeneration function of Mg/Al layered double hydroxide nanoparticles. These nanoparticles were found to upregulate the expression of TGF-β receptor 2 and TGF-β2 to reduce inflammation, thus accelerating axonal regrowth (Zhu et al., 2021). *In vivo* research further suggested that TGF-β1 coordinated with adipose-derived mesenchymal stem cells to enhance nerve regeneration by inhibiting inflammatory responses (Luo et al., 2012). By functioning as an anti-inflammatory regulator on innate and adaptive immune cells, TGF-β can help maintain the homeostasis of the local microenvironment and promote neural regeneration.

#### Macrophage Polarization

After PNI, apart from the function of removing the myelin debris, the infiltrated macrophages are educated by the local injured microenvironment and are polarized to an anti-inflammatory M2 phenotype, thus promoting peripheral nerve regeneration (Ferrante and Leibovich, 2012; Chen et al., 2015c; Liu P. et al., 2019). As discussed above, M2 macrophages support axonal regeneration, and TGF-β polarizes the macrophages recruited to the site of injury to the M2 phenotype. During the infiltration of macrophages, the expression level of TGF-β was shown to increase progressively and peak at day 7 after injury (Rotshenker, 2011), indicating that its higher levels persist longer than those of IL-6 and IL-10 (van Rossum et al., 2008). Therefore, macrophages are exposed to TGF-β for a relatively long time after nerve injury. The ability of TGF-β to polarize macrophages into the M2 phenotype is well demonstrated. In a study, TGF-β was found to activate the Akt/FoxO1 pathway in pro-inflammatory M1 macrophages, thus polarizing them into the M2 phenotype (Liu F. et al., 2019). Besides, M2 macrophages can also be induced *via* TGF-β1 signaling by suppressing the NF-κB-dependent cytokine/chemokine-producing pathway in macrophages (Echeverry et al., 2013). Interestingly, after being transformed by TGF-β, these M2 macrophages secrete sufficient amounts of TGF-β in the neuroinflammation environment and further polarize more M1 macrophages into the M2 phenotype, making this polarization a positive feedback process (Sulaiman and Gordon, 2009; Shapouri-Moghaddam et al., 2018).

#### Modulation of T Cells

T lymphocytes constitute approximately 11% of all immune cells that infiltrate the site of injury in mice (Echeverry et al., 2013). Findings of an *in vitro* experiment indicate that TGF-β can induce the migration of both CD4+ and CD8+ T lymphocytes to the site of injury (Adams et al., 1991). TGF-β1 inhibits the function of effector T cells and antigen-presenting dendritic cells while maintaining the level of Treg cells (Strainic et al., 2013). Notably, Treg cells supported by TGF-β are found to have neuroprotective roles, producing anti-inflammatory mediators such as IL-4, IL-10, and TGF-β1 to alleviate the local inflammatory response (Echeverry et al., 2013). Apart from Treg cells, TGF-β attracts Th2 cells that secrete IL-4 and IL-10 to control pro-inflammation and bias for a Th2-supporting environment due to its persisting level in the injured nerves, and consequently improves nerve recovery (van Rossum et al., 2008; Wang et al., 2020).

### Effects on Neurotrophic Factors

NGF and GDNF are considered key neurotrophic factors in PNS, and TGF-β can regulate their effects on promoting neural regeneration. NGF is immediately produced in SCs and fibroblasts after the injury, and its mRNA expression increases at the injury site and further distal to it (Rotshenker, 2011). TGF-β1 increases rapidly after injury and increases NGF expression (Yokozeki et al., 2021). TGF-β1 is synergistic with the trophic effects of NGF on neuronal survival and fundamental functions. In neonatal dorsal root ganglion neurons combined with TGF-β, although neuronal survival and levels of the substance P expressed per neuron were dramatically increased, NGF expression levels remained unchanged, indicating the synergy of TGF-β and NGF (Chalazonitis et al., 1992). These data suggest that TGF-β can facilitate NGF in terms of both its expression level and neurotrophic functions.

Many studies have shown that GDNF requires the presence of TGF-β to fully exert its trophic potential both *in vitro* and *in vivo* (Krieglstein et al., 1998; Sulaiman, 2016). Without TGF-β, GDNF is observed to have no neurotrophic effect when cultured with neurons. This special connection between TGF-β and GDNF on neuron survival is mediated by the activation of PI3 kinase, and therefore a specific PI3 kinase inhibitor is able to completely abolish its trophic effects. TGF-β synergizes with GDNF by protecting glycosylphosphatidylinositol (GPI)-linked receptors. After phosphatidylinositol-specific phospholipase C-mediated hydrolysis of GPI-anchored receptors, TGF-β is shown to stabilize and recruit the GPI-linked GDNF-α receptor (Krieglstein et al., 1998), thus allowing GDNF to exert its neurotrophic effects on neurons. Corroborating these findings, another research has suggested a similar result that TGF-β3 permits GDNF signaling and its neurotrophic effects by inducing the clustering of GDNF-α receptor 1 in lipid microdomains on the plasma membrane. Nevertheless, this effect is mediated by the activation of ERK/MAPK instead of the PI3K signaling pathway (Peterziel et al., 2002). These studies have added clarity to our understanding of the mechanisms underlying the permissive effects of TGF-β on GDNF.

## Regeneration

### Schwann Cell Proliferation and Apoptosis

During peripheral nerve development, TGF-β regulates the proliferation and apoptosis of SCs to maintain the proper quantity of them. Similarly, at different stages of neural regeneration, proliferation and apoptosis occur in SCs and play a critical role in establishing the proper axon-SC ratio, which is crucial for axons before their myelination (D’Antonio et al., 2006; Li et al., 2015). Notably, SC proliferation appears to be dispensable for nerve regeneration. A study suggests that axonal regeneration and myelination occur normally even without distal SC proliferation because the key factor for SCs to promote regeneration is their contact with axons instead of their number. Surplus SCs without axonal contact are removed from the nerve, making the number of SCs align precisely with the number of axons (Yang et al., 2008).

TGF-β can promote both proliferation and apoptosis in SCs. Research has also shown that TGF-β1 regulates both SC proliferation and apoptosis during Wallerian degeneration after nerve injury (Li et al., 2015). Blocking the TGF-β signaling pathway inhibits injury-induced proliferation and apoptosis of SCs, whereas TGF-β1 overexpression enhances both of these activities (D’Antonio et al., 2006; Li et al., 2015). Different TGF-β1 expressions affect Smad and AKT pathways, but do not affect c-Jun or ERK pathways, indicating that TGF-β1 regulates SC survival and death by the former pathways (Li et al., 2015).

Nevertheless, the effects of TGF-β on SCs strongly depend on different contexts. Treatment of SCs with TGF-β1 alone has no significant cytotoxic effect, but treatment with a combination of TGF-β1 and TNF-α leads to programmed cell death (Luo et al., 2021). TGF-β1 can kill non-myelinating SCs in the distal stump of the injured developing nerves but not SCs that express myelin proteins in normal nerves (Parkinson et al., 2001). This is because TGF-β phosphorylates c-Jun on the serine-63 residue and activates AP1-dependent transcription in naïve SCs, which induces SC death *via* the JNK/c-Jun signaling pathway. It is observed that caspase-3 is activated in SCs, indicating that these cells die by apoptosis. In contrast, decreasing the ability of TGF-β to phosphorylate c-Jun in differentiated SCs makes them less vulnerable to death (Parkinson et al., 2001). Furthermore, although TGF-β induces apoptosis in the absence of neuregulin-1, it promotes proliferation in the presence of neuregulin-1 (Parkinson et al., 2001; D’Antonio et al., 2006). Taken together, the influence of TGF-β on SCs is context-dependent, and this may be the underlying reason behind the maintenance of the proper number of SCs in the complex microenvironment following nerve injury.

### Formation of Bands of Büngner

**FIGURE 2:**
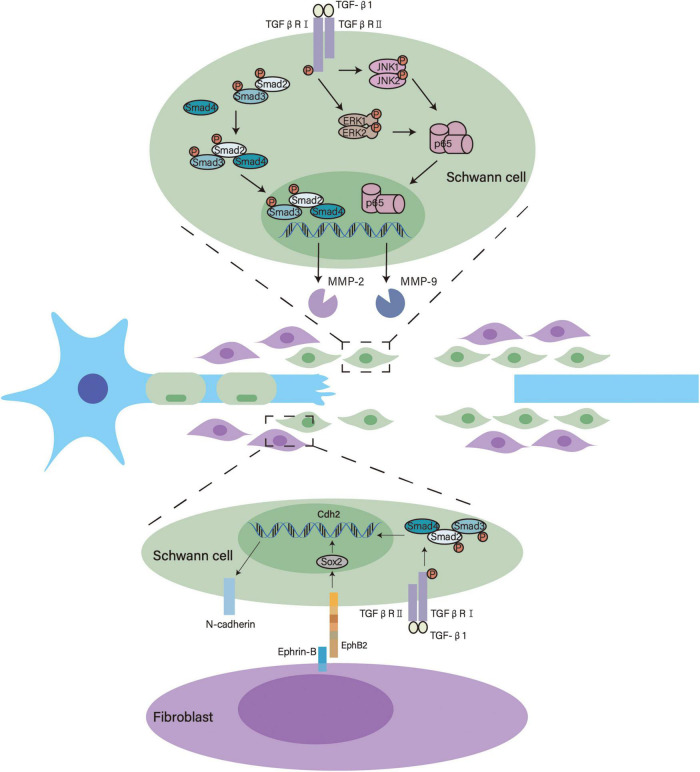
Underlying mechanisms of TGF-β in the formation of bands of Büngner. TGF-β activates Smad2/3 and enhances MMP-2 transcription. Meanwhile, TGF-β activates ERK1/2 and JNK1/2 that modulate p65/NF-κB, thus promoting MMP-9 transcription. These signaling pathways facilitate SC migration and invasion. Furthermore, TGF-β interacts with Eph signaling and upregulates the expression of N-cadherin to induce SC sorting.

After nerve injury, SCs form Bands of Büngner through which axons regenerate and extend (Min et al., 2021). This structure provides guidance for regrowing axons and increases the speed of axonal regeneration, thus making it an important basis for neural regeneration (Bearce et al., 2015; Min et al., 2021). The formation of Bands of Büngner involves SC migration, invasion and alignment in the nerve bridge, where TGF-β participates and facilitates this process (Figure 2). TGF-β is abundantly present in the nerve bridge. Even in the absence of macrophages, the sciatic nerve was found to produce an appreciable amount of TGF-β by itself after an injury (van Rossum et al., 2008).

TGF-β enhances the migration and invasion of SCs in the nerve bridge by transforming their phenotypes. SCs in the nerve bridge show more pronounced invasive and mesenchymal characteristics than SCs in the distal stump. The mesenchymal traits of SCs in the nerve bridge are closely associated with TGF-β, this is substantiated by the TGF-β signaling pathway being highly activated in these cells and exogenous TGF-β contributing to SC invasiveness (Clements et al., 2017). Using RSC96 SCs, Muscella et al. showed that TGF-β1 activates MMP2 *via* the canonical SMAD2/3 signaling pathway and MMP9 *via* the ERK1/2–JNK1/2–NF–κB pathway to significantly enhance the migratory and invasive behaviors of SCs (Muscella et al., 2020).

Deformation is another vital origin of SC motility. It is reported that SCs extend and retract leading processes to migrate (Min et al., 2021). These processes can be seen in SCs after 4 h TGF-β1 treatment. The underlying mechanism could be that the upregulation of β1 integrin makes these cells more acutely detect migratory cues in the local environment (Rosner et al., 2005).

After migration and invasion into the nerve bridge, TGF-β also helps SCs form multicellular cords. During the formation of bands of Büngner, fibroblasts come into direct contact with SCs. EphrinB2 expressed by fibroblasts activates EphB2 on SCs, which triggers the relocalization of N-cadherin in SC junctions. This interaction switches the behavior of SCs from repulsion to adhesion and orchestrates organized cell migration, thus facilitating the formation of bands of Büngner (Parrinello et al., 2010). On one hand, TGF-β upregulates the expression of Cdh2 in SCs through the canonical Smad signaling pathway, increasing the expression of N-cadherin; on the other hand, TGF-β also promotes the proliferation of fibroblasts that carry EphrinB2 (Parrinello et al., 2010; Kim et al., 2013; Frangogiannis, 2020). Therefore, TGF-β may participate in the sorting of SCs into cords that mechanistically guide axonal regrowth.

### Direct Regeneration-Promoting Effects on Neurons

Thus far, we have discussed the role of TGF-β in facilitating a regeneration-permissive environment. Additionally, TGF-β is found to exert positive effects on neuronal intrinsic growth capacity as well. The intrinsic growth capacity of neurons relies on the upregulation of regeneration-associated genes (RAGs) (Chandran et al., 2016). RAGs are a series of neuron-intrinsic genes that are activated by the axotomy of peripheral nerves. As they are involved in various pathways instrumental in beginning the regeneration program, they are indispensable to successful neural regeneration (Fawcett and Verhaagen, 2018). The activation of RAGs is associated with the switch of a neuron from a transmitting to a regrowing state (Sulaiman and Nguyen, 2016). However, this upregulation of RAGs is relatively transient, and the expression of RAGs wanes completely by 4–6 weeks after nerve injury (Sulaiman and Dreesen, 2014). Experiments have shown that TGF-β treatment stimulates and prolongs the expression of RAGs, thus improving axonal regeneration after chronic nerve injury (Sulaiman and Dreesen, 2014). This can perhaps explain the ample evidence on the direct regeneration-promoting effects of TGF-β. Neurons treated with TGF-β1 activate the canonical TGF-βRI/ALK5 pathway, resulting in improved neuronal survival and neurite outgrowth even in a growth-inhibitory environment. Conversely, blocking TGFβRII leads to a reduction in the length of regrowing axons (König et al., 2005). Treatment with TGF-β1 significantly increases the survival and the length of neurites extended by retinal ganglion cells, which is concomitant with increased expression of NF-160 (a structural component of the neuronal cytoskeleton) and UCH-L1 (a neuron-specific deubiquitinating enzyme). In this TGF-β-induced process, p38 MAPK but not Smad signaling pathways is required, thus indicating the role of Smad-independent pathways in the function of TGF-β (Walshe et al., 2011). Further studies suggest the presence of more mechanisms underlying the effect of TGF-β on neuronal intrinsic growth capacity. Mechanistically, after the activation of the TGFβRII/ALK5 signaling axis, protein kinase A phosphorylates the E3 ubiquitin ligase SMURF1, inducing a switch of its preferred substrate from PAR6 to Ras homolog A. Accordingly, TGF-β may enhance neurite outgrowth at the cytoskeleton level (Kaiser et al., 2020).

## The Potential of Exogenous Transforming Growth Factor-β in the Therapeutic Application

As numerous basic studies have reported on the pro-regenerative effects of TGF-β in various cellular responses in peripheral nerve regeneration, the translation of this knowledge into clinical practice for treating PNI is attractive. Table 1 presents some of the findings from research on exogenous TGF-β and its therapeutic applications.

**TABLE 1:** Findings of exogenous transforming growth factor-beta and its effects on nerve repair.

Animal	Surgical method	Administration	Findings	References
The Sprague–Dawley rats	Sciatic nerve transection and a 10 mm gap is made	Chitosan/gelatin-based nerve guide combined with 100 ng/ml TGF-β1 and 1 × 10^6^ cells/ml SCs	The recovery of nerve conduit with TGF-β and SCs is better than those treated with nerve graft alone	Nie et al., 2014
Dogs	Sciatic nerve transection and 50 mm nerve segment removed	100 ng/ml TGF-β1 is injected into the XANM nerve graft and both stumps of nerve gap are bridged with tissue-engineering nerve	TGF-β1 combined with ADSCs is able to promote long-nerve regeneration	Luo et al., 2012
Adult female Sprague–Dawley rats weighing about 200 g	Tibial nerve transection	Gelfoam soaks the 2 ng/mL forskolin plus 0.5 μM TGF-β solution for 10 min and is wrapped around the injury nerve	Single local application of TGF-β plus forskolin reactivates SCs and macrophages, increases regeneration-associated proteins, and promotes axonal regeneration	Sulaiman et al., 2018
Rats	Chronic denervation of common peroneal nerve before tibial-common peroneal nerve cross-suture	TGF-β (dose not given)	TGF-β reactivates chronically denervated SCs and supports neural regeneration	Sulaiman and Gordon, 2009
The Sprague-Dawley rats weighing about 200 g	Tibial nerve transection	Gelfoam containing 0.5 μM TGF-β and 2 ng/ml forskolin wrapped around the nerve	Treatment of TGF-β plus forskolin promotes axonal regeneration by increasing expression of growth-supportive genes and reactivating SCs and macrophages at the lesion site	Sulaiman and Dreesen, 2014
Adult female Sprague-Dawley rats	Tibial-common peroneal nerve cross-suture	Nerve explants are left in 1 ng/ml TGFβ and 0.5 μM forskolin before in vivo experiments	TGF-β and forskolin rescue long-term chronically denervated SCs to support axonal regeneration	Sulaiman and Gordon, 2002
Adult male Sprague–Dawley rats weighed 250–275 g	Partial sciatic nerve ligation of the common sciatic nerve	Intraneural injection of 0.1 μg, 0.5 μg or 1.0 μg recombinant TGF-β1 to the injury site	TGF-β1 delays and attenuates neuropathic pain without delaying nerve regeneration by modulating local immune cells	Echeverry et al., 2013
Adult male CD1 mice (25–32 g)	Tibial and common peroneal nerves transection	Intrathecal injection of 2 or 10 ng/ml TGF-β1	Exogenous TGF-β1 attenuates neuropathic pain	Chen et al., 2015a
				

As mentioned above, TGF-β enhances neural regeneration by local addition. After chronic nerve injury, TGF-β plus forskolin improves axonal recovery by upregulating RAGs and activation of SCs and macrophages (Sulaiman and Gordon, 2009; Sulaiman and Dreesen, 2014; Sulaiman and Nguyen, 2016; Sulaiman et al., 2018). TGF-β can also attenuate nerve injury-induced hypersensitivity. Intraneural injection of TGF-β1 can delay and relieve neuropathic pain by decreasing MAC1+ macrophages and T-lymphocyte infiltration (Echeverry et al., 2013). Both extrinsic TGF-β1 and intrathecal injection of bone marrow stromal cells that secrete TGF-β1 can inhibit neuropathic pain (Chen et al., 2015a). However, in actual practice, the direct application of biochemical factors at the injury site is not enough, because the physical condition is complex and it is difficult to ensure adequate local concentration of these factors during nerve regeneration. Nerve graft is a traditional repairing strategy and is considered the gold standard treatment for nerve regeneration (Carvalho et al., 2020). A study shows that overexpression of TGF-β1 in human amniotic mesenchymal stem cells markedly prevents xenograft rejection in mice (Chai et al., 2019).

Nerve tissue engineering has emerged as a constructive strategy for PNI treatment. Nerve guidance conduits (NGCs), the core of nerve tissue engineering, have gained attention recently. Research indicates that NGCs combined with cells (e.g., SCs, macrophages, and stem cells) and neurotrophic factors are highly effective in promoting axonal growth. This combination (NGCs, cells and stimuli) serves both as a scaffold and a carrier of molecular and mechanical signals in the process of regeneration, thus offering a prospective method of peripheral nerve repair (de Assis et al., 2022). SCs are the most used cells in NGCs to make the biomaterial regrow axons faster and more effectively (Berrocal et al., 2013). Research shows that a combination of TGF-β, NGF and neuregulin evidently enhances SC alignment in oriented collagen (Ribeiro-Resende et al., 2009). Chitosan/gelatin conduits combined with TGF-β and SCs significantly improve nerve repair in comparison with chitosan/gelatin conduits alone, and they even offer functional recovery comparable to that obtained with autografts (Nie et al., 2014). M2 macrophages are also included in the conduits to improve regeneration (Chen et al., 2015c). As summarized above, TGF-β is an anti-inflammatory factor that polarizes macrophages into the M2 phenotype, and a critical mediator of interaction between macrophages and SCs. Thus, TGF-β can be used in NGCs to promote the polarization of macrophages into the M2 phenotype. Difficulties in obtaining and cultivating SCs limit their direct use, and therefore, stem cells can act as a source of SC-like cells in NGCs (de Assis et al., 2022). Notably, TGF-β is also found to improve the survival and pro-regenerative capacity of stem cells. Upon TGF-β1 exposure, adipose stem cells in the acellular nerve matrix show enhanced vascular endothelial growth factor-dependent angiogenesis that helps protect neurons and reduces apoptosis (Luo et al., 2012). Human Wharton’s jelly-derived mesenchymal stem cells applied to injured sciatic nerves of mice have been shown to promote regeneration and functional recovery. The high level of TGF-β that induce Treg cells differentiation are crucial to this outcome (Wang et al., 2020).

Despite numerous positive effects of TGF-β in PNI experimental treatments, several studies show that it can have negative impacts on recovery (Li et al., 2017). Some studies find that TGF-β antibody enhances axonal regeneration by effectively reducing collagen production and scar formation (King et al., 2004; Taskinen et al., 2004). This phenomenon can be partly explained by the distinct expression and effects of TGF-β in the endoneurium and epineurium. In the endoneurium, TGF-β1 can support axonal regrowth, whereas in the epineurium it can be more harmful for regeneration and may contribute to epineurial scarring and neuroma formation (Taskinen et al., 2004). Thus, the site of TGF-β treatment greatly affects its effects when locally applied. Another research has shown that TGF-β1 inhibits the outgrowth of sensory neurons derived from the ND7/23 cell line by regulating the GSK-3β/CRMP-2 loop *via* both canonical and non-canonical signaling pathways. This can offer another possible reason for the negative impacts of TGF-β on regeneration (Jeon and Huxlin, 2020).

Overall, further research is required to ensure the safety and effectiveness of the application of TGF-β in neural regeneration, and eventually obtain new therapeutic perspectives for PNI.

## Conclusion and Perspective

**FIGURE 3:**
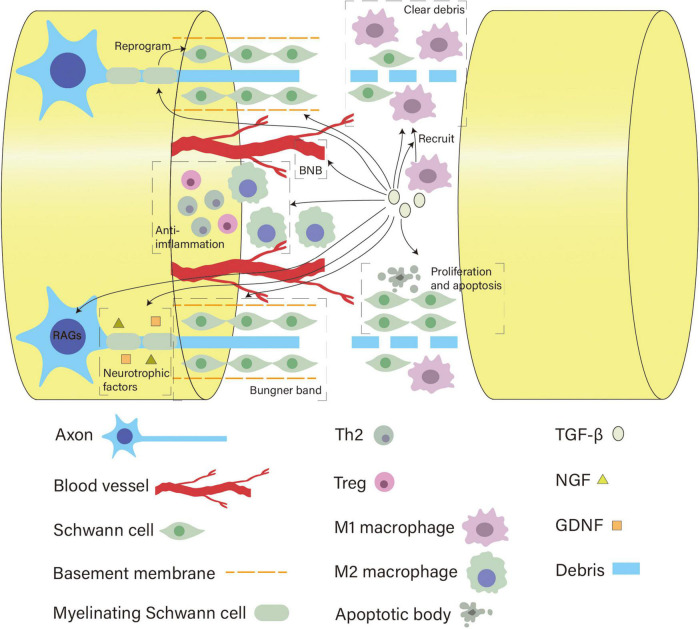
Overview of the diverse roles of TGF-β in neural regeneration. After nerve injury, TGF-β helps recruit macrophages and enhances the capacity of macrophages and SCs to clear myelin debris by upregulating the expression of MerTK receptors and MMPs, respectively. Meanwhile, it initiates reprogramming of SCs, regulates their number, protects the basement membrane, and promotes their migration and alignment to form bands of Büngner. TGF-β can act on pericytes and tight junction proteins to modulate the BNB permeability to regulate the local injury milieu. As a potent anti-inflammatory cytokine, it polarizes M1 macrophages into the M2 phenotype and sustains Tregs and Th2 cells to create a growth-supportive environment. In addition, TGF-β promotes neuronal growth capacity by activating RAGs of neurons and facilitating the neurotrophic effects of NGF and GDNF.

As a pleiotropic cytokine with neuroprotective functions, TGF-β is a promising target for promoting nerve regeneration and recovery after PNI. Various cells implicated in peripheral nerve repair such as macrophages, SCs and neurons, can secrete and respond to TGF-β. Although the exact mechanisms behind peripheral nerve regeneration remain to be fully elucidated, current literature has already focused on some specific biological processes that are considered crucial to this process (e.g., Wallerian degeneration). In this review, we conclude that TGF-β can guide these critical processes toward a regeneration-promoting direction by regulating the behavior of major cells involved (summarized in Figure 3). Additionally, TGF-β applied to tissue engineering shows encouraging outcomes, further indicating its role in peripheral nerve regeneration.

In recent years, with the development of tissue engineering, NGCs have become a hot topic in the field of peripheral nerve regeneration. Combined with cells and neurotrophic factors, NGCs can provide physical support and mimic the regenerative microenvironment of PNS, thereby greatly promoting the regenerative effects. TGF-β is abundantly present in the natural nerve bridge and participates in multiple regeneration processes. It can help optimize the interaction between cells in the scaffold and enhance its regenerative capacity. However, the role of TGF-β in NGCs is rather neglected. It has seldom been included in the currently available design of NGCs, unlike other factors such as IL-4, brain-derived neurotrophic factor and GDNF (reviewed by Li et al., 2021). This is probably because its application has some problems that remain to be solved. For example, the proper concentration and timing of TGF-β addition remain unknown. TGF-β may exert adverse effects on the host’s immune response. TGF-β has oncogenic properties, and its signaling pathway is an important pathogenic mechanism in tissue fibrosis (Hu et al., 2018). It supports axonal regrowth but may also deter regeneration by inducing epineurial scarring and neuroma. Further studies are needed to elucidate the precise mechanisms of peripheral nerve regeneration and the role of TGF-β signaling pathways in it to overcome these limitations. Embedding TGF-β in nerve tissue engineering technology like NGCs is a future direction to further explore its potential in nerve regeneration. In PNS, TGF-β can reactivate chronically denervated SCs and regulate macrophages to support axonal regeneration. Future studies can also explore how these cellular techniques can be used to improve regeneration after spinal cord injury. Although increasing evidence demonstrates the effects of TGF-β in peripheral nerve regeneration, there is still a long way to go before these encouraging results of basic research can be translated into clinical practice.

## Author Contributions

ZY and JW contributed to design, drafted, and critically revised the manuscript. CZ and JH contributed to conception, design and critically revised the manuscript. All authors read and approved the final manuscript.

## Conflict of Interest

The authors declare that the research was conducted in the absence of any commercial or financial relationships that could be construed as a potential conflict of interest.

## Publisher’s Note

All claims expressed in this article are solely those of the authors and do not necessarily represent those of their affiliated organizations, or those of the publisher, the editors and the reviewers. Any product that may be evaluated in this article, or claim that may be made by its manufacturer, is not guaranteed or endorsed by the publisher.

## Abbreviations

BNB: blood nerve barrier
ECM: extracellular matrix
GDNF: glial cell line-derived neurotrophic factor
GPI: glycosylphosphatidylinositol
IL: Interleukin
LAP: latency associated peptide
LRRC33: leucine-rich repeat-containing protein 33
L1-CAM: L1-cell adhesion molecule
MMP: matrix metalloproteinase
NF-160: neurofilament-M
N-CAM: neural-cell adhesion molecule
NGF: nerve growth factor
NGCs: nerve guidance conduits
PNI: peripheral nerve injury
RAGs: regeneration-associated genes
SCs: Schwann cells
TGF- β: transforming growth factor- β
TIMP-1: tissue inhibitor of metalloproteinases-1
UCH-L1: ubiquitin carboxy-terminal hydrolase L1.
